# Chairside Screening for Undiagnosed Diabetes and Prediabetes in Patients with Periodontitis

**DOI:** 10.1155/2022/9120115

**Published:** 2022-05-28

**Authors:** Silvia Masiero, Alice Alberti, Stefano Corbella, Luca Francetti

**Affiliations:** ^1^Department of Periodontology, Università degli Studi di Torino, Turin, Italy; ^2^Department of Biomedical, Surgical and Dental Sciences, Università degli Studi di Milano, Milan, Italy; ^3^IRCCS Istituto Ortopedico Galeazzi, Milan, Italy; ^4^Department of Oral Surgery, Institute of Dentistry, I. M. Sechenov First Moscow State Medical University, Moscow, Russia

## Abstract

**Introduction:**

Globally, it is estimated that half of all people with diabetes are undiagnosed. Because of the well-documented bidirectional link between diabetes and periodontitis, dentists and dental hygienists may have the possibility to screen a targeted population for diabetes during routine dental visits. The aim of the present study is to investigate the effectiveness of one device for diagnosis of diabetes/prediabetes used in one private dental practice and investigate the correlation between the levels of HbA1c and periodontal parameters.

**Methods:**

Periodontal patients that were never diagnosed with diabetes were asked to fill a risk assessment questionnaire for type 2 diabetes mellitus. PD, CAL, FMBS%, FMPS%, and HbA1c through a prick-finger test were measured before and after periodontal therapy or only once in patients following supportive periodontal therapy.

**Results:**

A total of 98 subjects were screened, and among them, one had diabetes and 30 had prediabetes. The mean value of HbA1c was 5.62% for patients with untreated periodontitis and 5.42% for periodontally treated patients. The diagnosis of diabetes resulted to be correlated to FMBS% and FMPS%, while HbA1c levels were correlated to FMBS%, FMPS%, and periodontitis grade.

**Conclusion:**

The present chairside diabetes-screening protocol allowed a consistent proportion of patients to become aware of their pathological or prepathological condition and to seek proper and timely medical care. Thus, dentists and dental hygienists could provide health promotion services and preventive measures.

## 1. Introduction

Diabetes mellitus (DM) is a metabolic disorder characterized by high blood glucose levels and alterations in the metabolism of carbohydrates, fats, and proteins, resulting from defects in insulin action and/or secretion [1]. The prevalence of diabetes has been increasing over the last decades. The last global estimates by the International Diabetes Federation (IDF) reported a prevalence of 10.5% in adults aged 20–79 years, for a total of 537 million people worldwide, and this value is expected to rise to 12.2% by 2045, reaching a total of 783 million affected people [2]. Subjects with a diagnosis of diabetes have an increased risk of developing a series of complications through generalized vascular damage affecting the heart, eyes, kidneys, and nerves. Moreover, diabetes was proven to be a known risk factor for periodontitis and for periodontal diseases in general so that it was considered a comorbidity of diabetes [3] and the sixth most frequent diabetic complication [4]. Such complications represent the first reason of the increased healthcare cost for diabetes: the global health expenditures for diabetes have been calculated to be more than 960 billion USD in 2021 [2]. In Europe, where 61 million people are affected by diabetes (with a prevalence of 9.2%), the economic burden of diabetes is 189 billion USD, accounting for 19.6% of the global diabetes-related expenditure. In Italy, which is the fifth European country for number of people with diabetes (4.5 million), an average cost of almost 3300 USD is spent for each subject with diabetes, and diabetes-related deaths reach 172 thousand [2].

It is estimated that globally almost half of all people living with diabetes are undiagnosed (44.7%), for a total of 239.7 million adults (20–79 years old), with the highest percentage in the Africa region (53.6%) and the lowest in North America (24.2%) [2]. The late diagnosis is often related to the insidious clinical onset of DM, which is typical type 2 diabetes mellitus (T2DM); however, also type 1 diabetes mellitus (T1DM) can have a progressive or intermittent development [5]. It must be noted that chronic complication could become evident even during the preclinical phase of the disease. For this reason, the screening for diabetes and prediabetes is of utmost importance for early detection, prevention, and timely intervention [6, 7]. The aforementioned estimates of undiagnosed diabetes indicate the urgent need for improved programs of diabetes screening. HbA1c is often used for this purpose, and it reflects average plasma glucose over the previous 8 to 12 weeks [8]. Prick-finger HbA1c is a simple test, which can be performed at any time of the day and does not require any special preparation such as fasting. For this reason, it is routinely used to assess glycemic control in patients affected by diabetes and it can be used as a screening test. Conversely, the WHO discouraged its use as a diagnostic test because of the issues regarding measurement accuracy [9].

Dentists and dental hygienists often visit patients frequently and regularly, and periodontal patients may represent an ideal target population for diabetes screening due to the extensively investigated bidirectional interplay between diabetes and periodontitis [10]. The two-way correlation between these pathologies is based on a complex of biological mechanisms that are not yet fully understood, as follows.

Diabetes, especially in cases with difficulties in controlling serum glucose levels, is correlated to an increased prevalence and severity of periodontitis [11–15]. Actually, the effects of diabetes on periodontal health do not depend on diabetes type, that is, its etiology but just on the levels of glycemic control [16].

Various mechanisms may be on the basis of the deleterious effects of DM on the periodontal health status, including periodontal microbial dysbiosis, the deficit of the cellular immunological response, and the alteration of the cytokines profile, with elevated levels of IL-1*β*, IL-6, RANKL/OPG, and other proinflammatory factors that may induce a hyperinflammatory state in periodontal tissues. A growing body of data suggested that hyperglycemia leads to high levels of advanced glycation end products (AGEs) and their receptors (RAGE) in gingival tissues, which can induce inflammation, oxidative stress, and alterations in the gingival fibroblast function. All these factors may contribute to aggravating periodontitis severity [17]. Moreover, some findings suggested that hyperglycemia may be related to impaired alveolar bone homeostasis: it has been reported that increased TGF-*ß* levels led to the suppression of osteogenic differentiation [18], and high levels of fatty acids induced osteoclastogenesis via TNF-*α*, leading to bone resorption [19].

On the other hand, periodontitis may determine a chronic overexpression of proinflammatory mediators (e.g., IL-1*ß*, IL-6, and TNF-*α*) which can induce the production of acute phase reactants by the liver (e.g., c-reactive protein and fibrinogen), lower the production of insulin in the pancreas, and lead indirectly to insulin resistance and hyperglycemia [20].

For the reasons outlined, screening periodontal patients during routine dental visits may help in early diagnosis of pathological or prepathological conditions [21, 22]. Previous studies have investigated diabetes screening in dental setting, with different methods of glycosylated hemoglobin A1c (HbA1c) assessment, clinical parameters measured, and patient selection criteria. [23–31].

The aim of the present study is to investigate the effectiveness of one device for diagnosis of diabetes/prediabetes used in one private dental practice and investigate the correlation between the levels of HbA1c and periodontal parameters.

### 1.1. Study Population and Methodology

#### 1.1.1. Participants

Consecutive periodontal patients visiting private dental practice (Saronno, Italy) from 1^st^ of July 2015 to 31^st^ of December 2020 were included in the present clinical study.

Periodontitis was defined according to the consensus report of the 2017 World Workshop on the classification of periodontal diseases [32]. As for subjects who received a diagnosis of periodontitis before the publication of the mentioned criteria, the diagnosis was confirmed by consulting clinical records and intraoral periapical radiographs. The following inclusion criteria were adopted: (1) diagnosis of periodontitis following the aforementioned criteria and (2) subjects aged 18 years old or above. Moreover, the following exclusion criteria were applied: (1) patients with incomplete or insufficient clinical data; (2) previously diagnosed diabetes; (3) pregnancy and/or lactation; (4) patients taking medication that may cause rapid glucose rise (e.g., steroids and antipsychotics) or affect HbA1c values (aspirin and antiretrovirals); and (5) medical conditions where HbA1c measurement is not indicated, according to the WHO recommendations [9]: patients with acute pancreatic damage, presence of genetic, hematologic, and illness-related factors that influence HbA1c and its measurement (e.g., haemoglobinopathies, certain anemias, chronic renal failure, alcoholism, and malaria).

### 1.2. Data Collection

During the visit, clinical parameters and the following full-mouth periodontal parameters were recorded, together with HbA1c levels: the probing depth (PD) and clinical attachment level (CAL) at six sites per tooth, full-mouth bleeding score (FMBS%), and full-mouth plaque score (FMPS%). Patients were also asked to fill in a questionnaire for T2DM risk assessment developed by the Finnish Diabetes Association [33], in which the following parameters were investigated: age range, body mass index (BMI), waist circumference, the level of physical activity, eating habits (frequency of fruit and vegetables consumption), assumption of medications for high blood pressure, previous episodes of high blood glucose (e.g., during pregnancy), and a family history of diabetes. Body weight, height, and waist circumference of the enrolled patients were also measured by the investigators.

Periodontal parameters were collected by two experienced periodontists through a periodontal probe with the University of North Carolina markings (UNC-15). PD, CAL, BOP, and PI were registered at six sites for each tooth; FMBS% and FMPS% were calculated. The number of missing teeth and implants was also registered.

HbA1c was measured through the prick-finger test using the Cobas® b101 *in vitro* diagnostic test system (Roche, Basel, Switzerland), which works by photometric transmission measurement. Subjects were asked to wash their hands with a nonalcoholic soap and dry them properly. A sterilized single-use needle was used to prick the palmar surface of the middle finger to produce a drop of blood, which was resorbed in the test disc and analyzed by using the diagnostic machine within 6 minutes. The value of HbA1c was displayed in the screen and expressed in % units and in mmol/mol units. If an HbA1c value of 5.7% or more was found, patients were asked to consult a medical doctor in order to confirm the diagnosis of diabetes through more accurate tests.

If the enrolled patients were following supportive periodontal therapy (SPT), the measurements of HbA1c levels and periodontal parameters were performed only once, while patients with untreated periodontitis were evaluated twice, namely, at the time of the diagnosis of periodontitis and after the completion of periodontal therapy. All procedures performed were in accordance with the 1964 Helsinki Declaration and its later amendments. The study was conducted with the human subjects' understanding and after getting signed a written informed consent.

### 1.3. Statistical Methods

The Shapiro–Wilk test was used to evaluate the normality of the distribution of the variables considered. Descriptive statistics were provided by means of the mean values and standard deviations for normally distributed variables. The prevalence of diabetes and prediabetes in patients undergoing periodontal maintenance therapy and their prevalence in patients with untreated periodontitis were compared.

Correlation between diabetic parameters (diagnosis of diabetes or prediabetes and levels of HbA1c) and periodontal parameters, including the periodontitis stage and grade and the results of the questionnaire was provided by the Pearson correlation test. The level of significance was *P* < 0.05. The statistical analysis was performed with software IBM® SPSS Statistics.

## 2. Results

A total of 98 subjects were screened, aged 55.8 ± 12.2 years (minimum 28, maximum 76 years), among which 48 were females and 50 were males. All of them were Caucasian except two who were Asiatic. Seventy-seven of them were undergoing supportive periodontal therapy, while 21 had untreated periodontitis. Among the latter, 14 were also evaluated after the completion of periodontal therapy, namely, two months to one year from the first screening.

Considering all 98 subjects, the initial screening test revealed one patient with diabetes, 30 with prediabetes, and 67 healthy patients. Measurements were performed by one operator in 54 cases and by another operator in 44 cases. As for the results of the type 2 diabetes risk assessment form by the Finnish Diabetes Association, the risk of developing T2DM within 10 years was low in 11 patients, slightly elevated in one, moderate in 27, high in 40, and very high in 19. The periodontitis stage was II in three patients, III in 68, and IV in 27, while grade was B in 25 patients and C in 73. All patients with an HbA1c value of 5.7% at the screening test received a confirmation of their diagnosis by the medical doctor, with 100% concordance.

As for patients who were taking part in a periodontal maintenance program (*n* = 77), no one had diabetes, 22 (28.6%) had prediabetes, and 55 (71.4%) were healthy. The mean levels of HbA1c were 5.41 ± 0.33% (4.7–6.4%). Among the 21 patients with untreated periodontitis, diabetes was diagnosed in 1 case (3.7%) and prediabetes in 8 cases (26.2%), and the mean values of HbA1c were 5.62 ± 0.64% (4.8–7.6%). The mean HbA1c value after periodontal therapy (*n* = 14) was 5.48 ± 0.41% (5.05–6.1%), with a mean decrease of 0.03 ± 0.22 from the baseline. Considering all patients with treated periodontitis (*n* = 91), that is, patients screened after periodontal therapy or during the maintenance phase, 26 (28.6%) found to be affected by prediabetes at the chairside screening test and 65 had normal HbA1c values, with a mean HbA1c of 5.42 ± 0.34% (4.7–6.4%).  Table 1 summarizes the results of HbA1c levels and periodontal parameters.

In all cases, the diagnosis of diabetes or prediabetes was confirmed by a medical specialist to whom the patient was referred.

HbA1c levels and the diagnosis of diabetes and prediabetes were positively associated with the total risk score resulting from the risk assessment questionnaire (*ρ* < 0.001, *ρ* = 0.018, and *ρ* < 0.001, respectively). As for periodontal parameters, the diagnosis of diabetes found to be correlated to FMBS% (*ρ* = 0.295 and *p*=0.003) and FMPS% (*ρ* = 0.251 and *p*=0.013) (Table 2). HbA1c levels were correlated to FMBS% (*ρ* = 0.211 and *p*=0.037), FMPS% (*ρ* = 0.216 and *p*=0.032), and periodontitis grade (*ρ* = 0.226 and *p*=0.025).

## 3. Discussion

The chairside screening performed in the present study showed its efficacy and effectiveness in identifying one pathological and a consistent number of prepathological conditions, with relevant advantages for the patients, in terms of secondary prevention of diabetes. Noticeably, all cases of diabetes or prediabetes that were individuated during the chairside screening test were confirmed by a specialist through further and more accurate tests after referral. Such a concordance could be considered as a confirmation of the usefulness of such a simple and inexpensive screening test beyond its limits. Considering the full concordance between the chairside screening and the diagnosis of the specialist, we decided to focus on the correlation between HbA1c levels and clinical periodontal parameters.

Although the statistical analysis showed a correlation between diabetes and FMBS% and FMPS%, it must be noted that this result relies only on one patient affected by diabetes, thus impairing its generalizability. This represents one limitation of the present study and indicates that larger samples are needed in future studies to validate more results of the present report. However, when observing the correlations between HbA1c levels and periodontal parameters, such a finding is confirmed: higher HbA1c levels were associated with higher FMPS%, FMBS%, and periodontitis grade. A possible explanation of these results is that all these parameters are strongly correlated with the inflammatory burden of the disease, and their effect on HBA1c levels can find biological plausibility in the previously mentioned bidirectional link between diabetes and periodontitis. On the one hand, even slightly elevated HbA1c levels seem to have a detrimental effect on the periodontium [34]; on the other hand, a growing body of data showed the influence of periodontitis on diabetic complications and incidences as updated in the joint workshop between the European Federation of Periodontology (EFP) and the IDF [35]. Data from 14 studies involving almost 32 thousand participants showed that periodontitis increased the risk of diabetic complications, including retinopathy (OR 1.2–2.8), renal, and cardiovascular complications. Moreover, it seems that periodontal patients exhibited a higher chance of developing T2DM (HR 1.19–1.33). As for metabolic control, studies about the effect of periodontal disease on glycemic control in patients with diabetes led to inconclusive results, but it was found that, in subjects without diabetes, periodontitis was associated with elevated levels of HbA1c, fasting blood glucose (FBG), and prediabetes prevalence compared to periodontally healthy subjects.

One further limitation regards the possibility of generalizing the results since the study included only a selected pool of periodontal subjects who had never been diagnosed with diabetes and prediabetes. Further studies with multiple cohorts of participants should be performed in the future in order to better evaluate the prevalence of undiagnosed diabetes in different pools of patients and thus identify more clearly the candidates during the chairside diabetes screening test.

Previous studies investigating chairside diabetes screening took into consideration various parameters to detect among dental patients and those with a higher risk of diabetes. These include age, morphometric parameters, high blood pressure, high cholesterol levels, and physical activity levels [24–30].

The possibility to perform diabetic screening in patients meeting the specific criteria about these parameters has been recommended also in a recent report by the Italian Association of Periodontology (Società Italiana di Parodontologia, SIDP) together with the two main national associations of diabetology (Associazione Medici Diabetologi, AMD and Società Italiana di Diabetologia, SID) [36]. In this document, it is recommended that dentists should perform diabetic screening in selected subjects who may be at a higher risk of diabetes, namely, (I) patients being >45 years old, showing no symptoms of diabetes, and not having their fasting blood glucose assessed in the previous 3 years and (II) patients with BMI ≥25 kg/m^2^ and presenting at least one of the indicated specific characteristics, including, among others, the first-degree family history of T2DM, physical inactivity, hypertension or antihypertensive drugs assumption, low levels of HDL cholesterol (<35 mg/dl) and/or high levels of triglycerides (>250 mg/dl), and polycystic ovary syndrome (PCOS). Moreover, dentists should prescribe a diabetology visit in patients with severe periodontitis and the first-degree family history of T2DM.

More generally, the European Federation of Periodontology (EFP) recommends that all dental patients without diabetes but exhibiting risk factors for T2DM, as assessed through the American Diabetes Association (ADA) questionnaire [37], should be informed of their condition and referred to a specialist.

Taking into consideration the limits of the present study, the great advantage of chairside diabetes screening must be underlined, as it made it possible for a consistent proportion of patients to become aware of their pathological situation or of their risk to develop diabetes and to seek proper and timely medical care.

Moreover, such a screening protocol does not require special training for health-care professionals, and it can be performed in a private practice dental office, which, since dental offices are widespread in the territory, could provide health promotion services and preventive measures for systemic health.

The role of dentists and dental hygienists in detecting and addressing health issues that are not strictly related to dentistry, but to health behaviors in general, has been extensively discussed and encouraged. This is particularly evident for smoking habit or improper eating habits, but we think that our attention should be focused also on other conditions such as diabetes also through screening protocols as the one proposed in our research.

In conclusion, the chairside diabetes screening protocol presented in our study allowed the identification of 31 subjects with pathological and prepathological condition, which were confirmed by a specialist with 100% concordance. The performed analysis on HbA1c and periodontal parameters revealed an association between HbA1c levels and FMBS%, FMPS%, and periodontitis grade, which should further be investigated.

## Figures and Tables

**Table 1:** Results of HbA1c levels and periodontal parameters expressed by means of frequencies or the mean ± standard deviation (min–max). For patients who repeated the screening after NSPT, differences between initial and final parameters are also reported.

	SPT patients (*n* = 77)	Patients screened before NSPT (*n* = 21)	Patients screened after NSPT (*n* = 14)	Total treated patients (*n* = 91)
Diabetes (diabetes, prediabetes, healthy)	0, 22, 55	1, 8, 12	0, 4, 10	0, 26, 65
HbA1c (%)	5.41 ± 0.33 (4.7–6.4)	5.62 ± 0.64 (4.8–7.6)	5.48 ± 0.41 (5.0–6.1) ∆ = 0,03 ± 0,22	5.42 ± 0.34 (4.7–6.4)
HbA1c (mmol)	35.69 ± 3.51 (28–47)	37.95 ± 7.14 (29–60)	36.50 ± 4.75 (31–44) ∆ = 0.36 ± 2.34	35.81 ± 3.71 (28–47)
Number of pockets between 5 and 7 mm	6.19 ± 6.79 (0–36)	23.62 ± 22.09 (1–93)	6.15 ± 8.63 (1–33) ∆ = 14.64 ± 15.50 (-60– 0)	6.18 ± 6.99 (0–36)
Number of pockets >7 mm	1.16 ± 2.08 (0–12)	7.33 ± 7.41 (0–34)	1.57 ± 2.21 (0–6) ∆ = -2,07 ± 3,15 (-11 – 0)	1.22 ± 2.09 (0–12)
Number of pockets >5 mm	7.35 ± 8.07 (0–39)	30.95 ± 27.17 (7–104)	7.29 ± 8.41 (1–33) ∆ = -22.36 ± 19,90 (-71 −1)	7.40 ± 8.05 (0–39)
Number of missing teeth	3.91 ± 4.48 (0–22)	3.57 ± 3.35 (0–12)	3.07 ± 2.92 (0–9) ∆ = 0,21 ± 0,43 (0–1)	3.81 ± 4,30 (0–22)
Number of implants	1.31 ± 2.55 (0–12)	0.57 ± 1.16 (0–4)	0.93 ± 1.54	1.25 ± 2.42 (0–12)
FMBS%	14.90 ± 13.34 (0–53)	59.33 ± 31.95 (7–100)	17.77 ± 14.31 (2–50) ∆ = -32,43 ± 26,60 (-81 –0)	15.31 ± 13.44 (0–53)
FMPS%	26.19 ± 13.98 (0–65)	64.10 ± 27.34 (10–100)	25.31 ± 7.09 (16–45) ∆ = -33.57 ± 29.39 (-77 – +12)	26.07 ± 13.18 (0–65)
Risk group	Low: 6 Slightly elevated: 0 Moderate: 24 High: 31 Very high: 16	Low: 5 Slightly elevated: 1 Moderate: 3 High: 9 Very high: 3	—	Low: 10 Slightly elevated: 1 Moderate: 26 High: 36 Very high: 18
Total risk score	9.70 ± 5.11 (0–24)	9.00 ± 4.66 (0–20)	—	9.55 ± 4.97 (0–24)
Periodontitis stage	Stage I: 0 Stage II: 3 Stage III: 53 Stage IV: 21	Stage I: 0 Stage II: 0 Stage III: 15 Stage IV: 6	—	Stage I: 0 Stage II: 3 Stage III: 64 Stage IV: 24
Periodontitis grade	A: 0 B: 22 C: 55	A: 0 B: 3 C: 18	—	A: 0 B: 24 C: 67

**Table 2:** Correlations between diabetes parameters and periodontal parameters.

		Periodontitis stage	Periodontitis grade	Number of pockets 5–7 mm	Number of pockets >7 mm	Number of pockets >5 mm	FMBS%	FMPS%	Extension	Number of implants	Number of missing teeth
Diabetes (*n* = 1)	*ρ*	0.155	0.059	0.045	0.078	0.057	0.295^*∗∗*^	0.251^*∗*^	0.161	−0.050	0.194
	Sign. (2-tailed)	0.129	0.561	0.659	0.445	0.579	0.003	0.013	0.114	0.623	0.056

Prediabetes (*n* = 30)	*ρ*	0.029	0.186	−0.123	−0.007	−0.100	0.067	0.125	0.021	0.042	0.005
	Sign. (2-tailed)	0.776	0.067	0.228	0.948	0.329	0.514	0.222	0.837	0.682	0.958

HbA1c levels (%) (*n* = 98)	*ρ*	0.124	0.226^*∗*^	−0.033	0.034	−0.017	0.211^*∗*^	0.216^*∗*^	0.027	−0.062	0.105
	Sign. (2-tailed)	0.222	0.025	0.749	0.738	0.869	0.037	0.032	0.792	0.541	0.304

*ρ* = Pearson correlation coefficient. ^*∗∗*^ Correlation is significant at the 0.01 level (2-tailed). ^*∗*^ Correlation is significant at the 0.05 level (2-tailed).

## Data Availability

The data used to support the findings of this study are included within the article. Individual data of all included patients are available on request after contacting the corresponding author.

## Additional Points

*Scientific Rationale for Study.* Periodontal patients may represent a selected pool of subjects for the screening of diabetes and prediabetes, which often remain undiagnosed for a long time.

*Principal Findings.* In the present study, a consistent proportion of patients became aware of their pathological or prepathological condition. A correlation was observed between HbA1c levels and FMBS%, FMPS%, and periodontitis grade.

*Practical Implications.* Dentists and dental hygienists may have an important role in the early identification of previously undiagnosed hyperglycemia through simple chairside diabetes screening protocols, thus making it possible to plan adequate prevention and treatment plans.
